# Reduced Binding of the Endolysin LysTP712 to *Lactococcus lactis* Δ*ftsH* Contributes to Phage Resistance

**DOI:** 10.3389/fmicb.2016.00138

**Published:** 2016-02-11

**Authors:** Clara Roces, Ana B. Campelo, Susana Escobedo, Udo Wegmann, Pilar García, Ana Rodríguez, Beatriz Martínez

**Affiliations:** ^1^DairySafe Group, Department of Technology and Biotechnology of Dairy Products, Instituto de Productos Lácteos de Asturias – Consejo Superior de Investigaciones CientíficasVillaviciosa, Spain; ^2^Institute of Food Research, Norwich Research ParkNorwich, UK

**Keywords:** phage resistance, *Lactococcus*, cell surface, endolysin, FtsH

## Abstract

Absence of the membrane protease FtsH in *Lactococcus lactis* hinders release of the bacteriophage TP712. In this work we have analyzed the mechanism responsible for the non-lytic phenotype of *L. lactis* Δ*ftsH* after phage infection. The lytic cassette of TP712 contains a putative antiholin–pinholin system and a modular endolysin (LysTP712). Inducible expression of the holin gene demonstrated the presence of a dual start motif which is functional in both wildtype and *L. lactis* Δ*ftsH* cells. Moreover, simulating holin activity with ionophores accelerated lysis of wildtype cells but not *L. lactis* Δ*ftsH* cells, suggesting inhibition of the endolysin rather than a role of FtsH in holin activation. However, zymograms revealed the synthesis of an active endolysin in both wildtype and *L. lactis* Δ*ftsH* TP712 lysogens. A reporter protein was generated by fusing the cell wall binding domain of LysTP712 to the fluorescent mCherry protein. Binding of this reporter protein took place at the septa of both wildtype and *L. lactis* Δ*ftsH* cells as shown by fluorescence microscopy. Nonetheless, fluorescence spectroscopy demonstrated that mutant cells bound 40% less protein. In conclusion, the non-lytic phenotype of *L. lactis* Δ*ftsH* is not due to direct action of the FtsH protease on the phage lytic proteins but rather to a putative function of FtsH in modulating the architecture of the *L. lactis* cell envelope that results in a lower affinity of the phage endolysin to its substrate.

## Introduction

Bacteriophages are obligate parasites that infect and, in most cases, eventually kill bacteria. After propagation inside the host, the new phage particles are ultimately released from the bacterial cell. Lysis of the host is a crucial step for survival of lytic phages and sophisticated mechanisms have evolved to maximize phage propagation. This is exemplified by the enrolment of several proteins dedicated to coordinate and time lysis such as holin/antiholins to disrupt the cytoplasmic membrane, endolysins to degrade the peptidoglycan layer, and spanins (in phages infecting Gram-negative hosts) required to overcome the outer membrane (Young, 2013).

Most dsDNA phages possess a typical holin–endolysin system, where a hydrophobic membrane protein, the holin, inserts into the cytoplasmic membrane forming holes. These holes are large enough to provide access to the cell wall for the endolysin that has up to this point been accumulating in the cytoplasm (revised by Catalão et al., 2013; Young, 2013). Endolysins with secretory signals, which are exported through the cytoplasmic membrane by the host general secretion system, have also been described. Lys44, the endolysin from *Oenococcus oeni* phage fOg44 has a typical cleavable signal peptide at its N-terminus (São-José et al., 2000). Others possess a SAR (Signal Arrest Release) domain through which the endolysin remains tethered to the membrane in an inactive form. In some cases, small hole-forming holins (or pinholins) insert into the membrane and dissipate the proton motive force. This signal triggers the release of the SAR domain from the membrane, prompting the endolysin to an activated state. Examples of these mechanisms have been described for phages P1 (Xu et al., 2004, 2005) and 21 (Park et al., 2006, 2007) of *Escherichia coli*, the phage ΦKMV of *Pseudomonas aeruginosa* (Briers et al., 2011) and more recently for the *Lactobacillus fermentum* phage φPYB5 (Guo et al., 2015). Bacterial cell lysis is a tightly regulated process and some phages synthesize a third protein, the antiholin, which inhibits oligomerization of holin molecules prior to hole/pore formation. Antiholins may be encoded by the same holin gene by virtue of a dual start motif or as an intragenic product (Bläsi and Young, 1996; Vukov et al., 2003; Park et al., 2006).

Endolysins from phages infecting Gram-positive hosts are often modular and characterized by the presence of one or more catalytic domains (CD) and cell wall binding domains (CBD). Enzymatic activities fall into different classes according to the specific bond that is cleaved within the peptidoglycan molecule. Amidase, glycosidase, endopeptidase, or lytic transglycosylase domains are commonly found in phage lysins. CBDs are responsible for the substrate recognition and the specificity of these peptidoglycan hydrolases (Fenton et al., 2010; Nelson et al., 2012; Schmelcher et al., 2012; Oliveira et al., 2013). In a recent survey, up to 13 distinct CBDs have been described within phage endolysins (Oliveira et al., 2013), encompassing species-, serovar-, or even strain-specific binding domains (Eugster et al., 2011) along with wide-spread carbohydrate binding moieties such as LysM present in proteins from all kingdoms (Mesnage et al., 2014).

We have previously reported that absence of the membrane protease FtsH renders the dairy starter *Lactococcus lactis* resistant to lysis by the temperate phage TP712 (Roces et al., 2013). TP712 is a member of the P335 group of lactococcal bacteriophages which belong to the *Siphoviridae* family and are most frequently found in the dairy environment. FtsH is an AAA+ (ATPase associated with various cellular activities)-type membrane protease conserved in eubacteria, mitochondria and chloroplasts which is involved in stress responses, protein quality control and regulation circuits acting as a protease or as a chaperone mainly on membrane proteins, although cytosolic substrates have also been identified (Ito and Akiyama, 2005; Langklotz et al., 2012). During infection of *L. lactis* Δ*ftsH*, TP712 virions were correctly assembled and retained infectivity but remained confined inside the cytoplasm, suggesting that the *ftsH* mutation might inhibit the late host lysis step of the phage lytic cycle (Roces et al., 2013). In this work, we aimed to explain the absence of lysis after phage propagation and investigated the functionality of the holin-endolysin system in *L. lactis* Δ*ftsH*. Our results rule out an active role of FtsH in triggering lysis. Instead, binding of the phage endolysin to the surface of *L. lactis* Δ*ftsH* cells is reduced, suggesting that a modified cell envelope is behind its non-lytic phenotype.

## Materials and Methods

### Microorganisms and Growth Conditions

All bacterial strains used in this study are shown in **Table 1**. *L. lactis* was routinely grown in M17 (Oxoid, Spain) supplemented with 0.5% glucose (GM17) at 30°C. When needed, chloramphenicol and erythromycin were added at 10 μg/ml. *Escherichia coli* was grown in 2xYT (Sambrook et al., 1989) at 37°C with vigorous shaking plus ampicillin (150 μg/ml) or chloramphenicol (50 μg/ml) as required. TP712 phage suspensions were prepared as previously described (Roces et al., 2013). *L. lactis* MG1363 Δ*acmA* TP712 lysogens were isolated from turbid lytic halos after spotting TP712 on *L. lactis* MG1363 Δ*acmA* lawns and subsequently colony-purified in GM17 plates. Lysogens were confirmed by lysis of the culture after induction with mitomycin C (MitC) (Sigma, Spain) and the presence of plaques after plating filtered supernatants on *L. lactis* NZ9000 (Roces et al., 2013). Prophage induction was carried out on exponentially growing cultures (OD_600nm_ = 0.2–0.3) with MitC at 1 or 2 μg/ml as indicated.

**Table 1:** Strains, bacteriophages, and plasmids used in this work.

	Description	Reference
***Lactococcus lactis***		
NZ9000	Wildtype, MG1363 *pepN::nisRK*. Host for nisin-inducible gene expression.	Kuipers et al., 1998
Δ*ftsH*	NZ9000, *ftsH* in frame deletion.	Roces et al., 2013
UKLc10	MG1363, *pepX, pepT, pepO, pepC, pepN::nisRK*	Wegmann et al., 1999
UKLc10 TP712	UKLc10 TP712 lysogen	Roces et al., 2013
UKLc10 Δ*ftsH* TP712	UKLc10 TP712 lysogen, *ftsH* in frame deletion	Roces et al., 2013
MG1363Δ*acmA*	Lacks the major autolysin AcmA	Buist et al., 1995
MG1363Δ*acmA* TP712	TP712 lysogen	This work
***Staphylococcus xylosus***		
CTC1642	Negative control fluorescence microscopy	M. Garriga, IRTA-Spain
***Escherichia coli***		
DH10B	Cloning host	Invitrogen
BL21CodonPlus(DE3)-RIL	Gene expression host. Cm^R^	Agilent Technologies
**Bacteriophages**		
TP712	Temperate phage. GenBank: AY766464	Roces et al., 2013
**Plasmids**		
pUK200	Nisin inducible P*_nisA_* expression vector. Cm^R^	Wegmann et al., 1999
pUK200::AH-H-6xHis	P*_nisA_*::*holTP712*-*His6* with functional ATG(1) and ATG(2)	This work
pUK200::AH-dH-6xHis	P*_nisA_*::ATG(2) to GGG *holTP712-His6*. Cm^R^	This work
pUK200::AH-dH	P*_nisA_*::ATG(2) to GGG *holTP712*. Cm^R^	U. Wegmann, IFR (UK)
pET21a	Inducible *E. coli* expression vector. Amp^R^	EMD Biosciences
pTRL1	Source of *mrfp* encoding mCherry fluorescent protein. Em^R^	García-Cayuela et al., 2012
pBL66	pET21a expressing mCherry-2xLysM_TP712_	This work

### General DNA Techniques

Restriction enzymes were purchased from TaKaRa (Japan). Ligations were carried out with T4 ligase (Fisher Scientific, Spain), for 14 h at 16°C in a final volume of 20 μl. Oligonucleotides were supplied by Macrogen (Korea). Chromosomal DNA was isolated with the GenElute Bacterial Genomic DNA Kit (Sigma, Spain). Plasmids were purified using the High Pure Plasmid Isolation Kit (Roche, Germany). DNA fragments were purified using the Ilustra GFX PCR DNA and Gel Band Purification Kit (GE Healthcare, UK). All constructions were checked by DNA sequencing by Macrogen (Korea).

### His-Tagged Holin Proteins Hol88TP712 and Hol74TP712

His-tagging of the holin proteins Hol74TP712 and Hol88TP712 was made by PCR using primers P_AH_FatI_F (5′ CAATCAGCATGCTCACGAAATTTACG 3′) and P_AH_6xHis_STOP_R (5′ TCAGTGATGGTGATGGTGATGTTTATTGTCTCCGTATCATTTGG 3′) at a final concentration of 0.3 μM, with Pwo SuperYield polymerase (Roche, Spain). The *holTP712* gene including the start codons ATG(1) and ATG(2) was amplified using 10 ng of chromosomal DNA of *L. lactis* UKLc10 TP712 as template. PCR conditions were: 93°C 2 min; 30 cycles of 93°C 15 s, 68°C 30 s, 72°C 30 s; 72°C 7 min. The PCR product was digested with FatI and ligated into pUK200 previously digested with NcoI/SmaI to yield the plasmid pUK200::AH-H-6xHis (**Table 1**). To obtain a DNA fragment where only ATG(1) was present, 10 ng of the plasmid pUK200::AH-dH (U. Wegmann, IFR, UK), in which ATG(2) has been replaced by the codon GGG (Gly), was used as DNA template. PCR conditions were 93°C 2 min; 35 cycles 93°C 15 s, 65°C 30 s, 72 °C 30 s; 72°C 7 min. The PCR product was cloned into pUK200 to yield pUK200::AH-dH-6xHis (**Table 1**) as described above.

*Lactococcus lactis* cultures (250 ml) with plasmids pUK200::AH-dH-6xHis and pUK200::AH-H-6xHis (**Table 1**), were induced with nisin (1.8 ng/ml) at an OD_600nm_ of 0.7 and grown at 30°C for 45 min. Cells were harvested by centrifugation (8000 *g*, 15 min, 4°C), resuspended in 25% sucrose and disrupted in a One Shot Cell Disrupter (Constant Systems LTD, UK). Cell debris was removed by centrifugation at 17000 *g*, 15 min, 4°C. Preliminary fractionation experiments revealed the presence of the His-tagged proteins in the membrane as well as in the cytoplasm fraction, likely due to the high gene expression levels. Subsequently, the proteins were purified from the latter fraction using the His Buffer Kit (GE Healthcare, UK) and Ni-NTA Suplerflow Resine (Qiagen, Germany) following manufacturer’s recommendations. His-tagged proteins were eluted with 0.5 M imidazol and resolved in tricine-SDS-PAGE 18%T-5%C gels (Schägger and von Jagow, 1987).

### Recombinant mCherry-2xLysM_TP712_ Fusion, Microscopy, and Cell Binding Assays

The *mrfp* gene encoding the red fluorescent mCherry protein was amplified using Phusion DNA polymerase (Fisher Scientific, Spain) with primers mrfp5 (5′ GTAGCTAGCGTTTCAAAAGGGGAGG 3′) and mrfp3 (5′ CGGGATCCTTTATATAATTCGTCCATGCC 3′) using the plasmid pTRL1 as template (**Table 1**) and 65 °C as annealing temperature. Similarly, the cell wall binding domain (CBD; 2xLysM_TP712_) of the TP712 endolysin was amplified with primers LysM5 (5′ ATGGATCCGGAAAACAGAAAGGCC 3′) and LysM3 (5′ CCGCTCGAGATAATTTAAAGTTTGACCAGC 3′) on *L. lactis* NZ9000. The *mrfp* PCR product was digested with NheI and BamHI and cloned into pET21a (**Table 1**). The resulting plasmid was digested with BamHI and XhoI and used to clone the 2xLysM_TP712_ PCR product digested with the same restriction enzymes to yield pBL66 (**Table 1**). Recombinant mCherry-2xLysM_TP712_ was overproduced in *E. coli* BL21CodonPlus(DE3)-RIL (Agilent Technologies, Spain) after induction of a 200 ml culture of exponentially growing (OD_600nm_ 0.4–0.5) cells with isopropyl-β-D-thiogalactopyranoside (IPTG) at 0.5 mM for 4 h at 37°C. Cells were disrupted by sonication and the soluble fraction purified with Ni-NTA Suplerflow Resin (Qiagen, Germany) following manufacturer’s recommendations. Elution buffer was exchanged to 100 mM NaCl, 50 mM NaH_2_PO_4_, 0.005% Tween 20, pH 8.0, using Zeba™ Desalt Spin Columns (ThermoFischer Scientific, Spain). Protein was quantified with Pierce BCA protein determination kit (ThermoFischer Scientific, Spain) and stored at 4°C in the presence of glycerol 50% (v/v). Binding assays were essentially performed as described (Schmelcher et al., 2010) in a Cary Eclipse fluorometer (Varian, Inc., Australia) using cell suspensions of exponentially growing cells of *L. lactis* NZ9000 and *L. lactis* Δ*ftsH*. Experiments were carried out with two independent cultures per strain and two technical replicates. The differences between the percentage of cell bound fluorescence shown by *L. lactis* NZ9000 and *L. lactis* Δ*ftsH* were tested for significance by use of the one-tailed Student *t* test. Results are expressed as means ± standard deviations. A *p*-value below 0.05 was considered statistically significant. For fluorescence imaging, a Nikon Eclipse 90i fluorescence microscope equipped with an appropriate filter set and a Nikon ACT-2U digital camera was used. *Staphylococcus xylosus* CTC1642 was also used as a control for unspecific binding.

### Treatment with Ionophores

*Lactococcus lactis* UKLc10 and *L. lactis* UKLc10 Δ*ftsH* TP712 lysogens were induced with MitC at 1 μg/ml and further incubated for 60 min at 30°C. Cells (50 ml) were washed with KKM buffer (50 mM potassium phosphate buffer, 50 mM KCl, 2 mM MgSO_4_, pH 6.4) and resuspended in 10 ml of KKM buffer plus 0.5% glucose. Centrifugation and washing steps were carried out at 4°C. Two-hundred microliter aliquots were transferred to microtiter plate wells containing 10 μl of 20 μM solutions of the ionophores nigericin (Sigma, Spain), valinomycin (Sigma, Spain) and nisin (Applin&Barret, UK), prepared in KKM buffer (final concentration 1 μM). The OD_600nm_ was followed at 30°C for 120 min in a Benchmark Plus Microplate Spectrophotometer (BioRad, USA). Non-MitC induced cultures were similarly manipulated and non-ionophore-treated cell suspensions were also analyzed as controls. Measures were made in triplicate.

### Protein Extracts and Zymograms

*Lactococcus lactis* MG1363 Δ*acmA* and *L. lactis* UKLc10 Δ*ftsH* TP712 lysogens were grown to OD_600__nm_ of 0.5 and the prophage induced with 2 μg/ml of MitC. Samples were collected at 0, 30, 60, 90 (onset of lysis) min and further at 120 and 180 min in the case of the Δ*ftsH* mutant. Cells were cooled on ice, collected by centrifugation (8000 *g*, 15 min, 4°C), washed with cold 50 mM sodium phosphate buffer pH 6.8 and concentrated in the same buffer at a cell density of 30 OD_600__nm_ units per ml. Cells (2 ml) were broken twice in the One Shot Cell Disrupter (Constant Systems LTD, UK). The insoluble fraction was pelleted by centrifugation at 4°C, 8000 *g* for 10 min and boiled for 3 min in 1/30 of the initial volume in SDS-PAGE loading buffer. For zymograms, SDS-PAGE gels were prepared incorporating either *L. lactis* NZ9000 or *L. lactis* Δ*ftsH* autoclaved cells to the gel matrix as previously described (Lepeuple et al., 1998). A total of 15 OD equivalents were loaded per lane. After electrophoresis, gels were washed three times with milliQ H_2_O for 10 min at room temperature and incubated overnight at 37°C, with gentle shaking, in 50 mM MES-NaOH pH 6.0, and 0.1% triton X-100. Gels were briefly washed with milliQ H_2_O and photographed without further staining with methylene blue.

## Results

### The TP712 Lysis Cassette Features a Holin Gene With a Functional Dual Start Motif and Both Holin Proteins are Synthesized in *L. lactis* NZ9000 and *L. lactis* Δ*ftsH*

**FIGURE 1:**
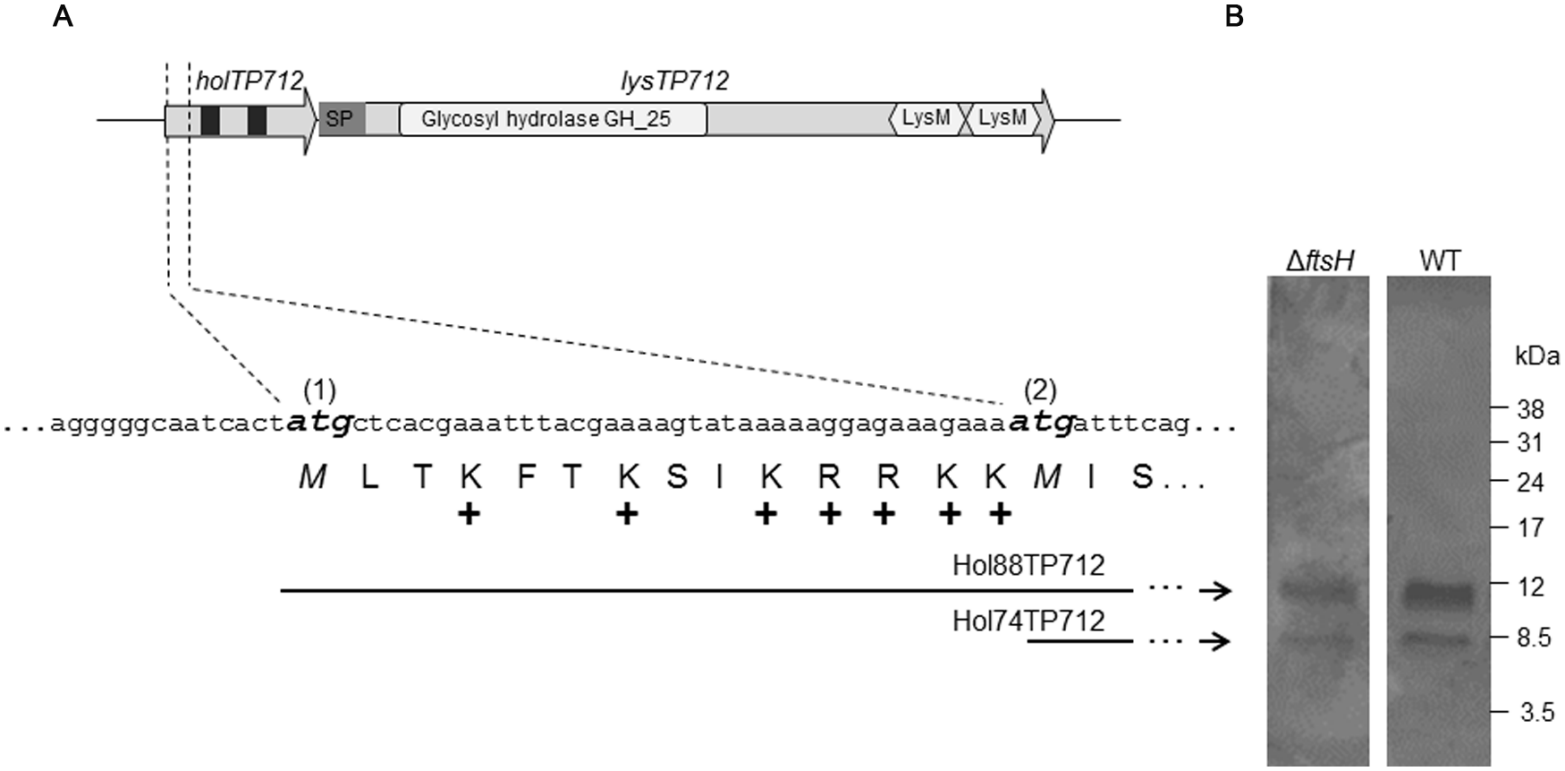
**The lysis cassette of the bacteriophage TP712. (A)** *holTP712* and *lysTP712* encode the putative holin/antiholin and endolysin LysTP712, respectively. The putative translational dual start motif in *holTP712* is shown in detail, where the ATG start codons (1) and (2) are shown in bold and italics. The holin proteins Hol88TP712 and Hol74TP712 are translated from ATG(1) and ATG(2), respectively. In *holTP712*,black squares indicate putative transmembrane domains (TMD). In *lysTP712*, the catalytic domain (CD; Glycosyl hydrolase GH_25) and cell binding domains (LysM) are indicated. SP: putative signal peptide. +: positively charged amino acid. **(B)** Hol88TP712 and Hol74TP712 are synthesized by both *L. lactis* NZ9000 (wildtype, WT) and *L. lactis* Δ*ftsH.* pUK200::AH-H-6xHis transformants were induced with nisin (1.8 ng/ml) for 45 min. His-tagged proteins were purified and resolved on a tricine SDS-PAGE gel. Molecular weight markers are shown on the right.

The lysis cassette of the phage TP712 comprises a putative pinholin gene *holTP712* (orf55) and the lysin gene *lysTP712* (orf56) (**Figure 1**). *holTP712* encodes a 74-aa membrane protein Hol74TP712 with two transmembrane domains (TMD), a topology that resembles that of the class II pinholin S^21^ of the *E. coli* phage 21 (Park et al., 2006). This protein would be synthesized from the start codon labeled as ATG(2) in **Figure 1**. However, another start codon, ATG(1), is located 42 nucleotides upstream from which another protein, a putative antiholin Hol88TP712 (88 aa), could be synthesized. Hol88TP712 would be endowed with an N-terminal extension of 14 amino acids, half of them positively charged (**Figure 1**).

In the case of the holin S^21^68 of phage 21, TMD1 has to translocate across the membrane to allow TMD2 to nucleate and form pinholes that will cause membrane depolarization and the subsequent activation of the SAR endolysin. This translocation event is retarded in the case of the antiholin S^21^71, due to an extra positive residue in its N-terminus. Since TMD1 acts *in trans* as a negative inhibitor of TMD2, S^21^71 delays formation of pinholes and regulates lysis of the host cell (Park et al., 2006; Pang et al., 2009, 2010). Assuming that a similar model may apply to TP712, we hypothesized that FtsH could regulate lysis timing by processing the N-terminus of Hol88TP712 to facilitate the translocation of TMD1, i.e., prompting the transition to the active holin topology.

To this end, plasmids pUK200::AH-H-6xHis and pUK200::AH-dH-6xHis were transformed into *L. lactis* NZ9000 and Δ*ftsH* to produce C-terminally His-tagged versions of both holin proteins or Hol88TP712-6xHis only, respectively. After nisin induction of pUK200::AH-H-6xHis, both Hol88TP712-6xHis, and Hol74TP712-6xHis proteins were synthesized, demonstrating that both ATG start codons were functional regardless of the genetic background of the host (**Figure 1**). Additionally, when the plasmid pUK200::AH-dH-6xHis was induced, only one protein with a molecular mass of 10412.5 Da was detected in both *L. lactis* wildtype and Δ*ftsH* (Supplementary Figure S1). The mass of this protein fits well with the theoretically expected mass for Hol88TP712-6xHis of 10413 Da. Thereby, under the assayed conditions, processing of the putative antiholin Hol88TP712 by FtsH could not be demonstrated.

### Membrane Depolarization of MitC-Induced *L. lactis* UKLc10 Δ*ftsH* TP712 Cells with Energy Poisons Does Not Accelerate Lysis

Ionophores have been previously used to simulate the action of pinholins in order to activate phage endolysins dependent on membrane depolarization (Xu et al., 2004; Nascimento et al., 2008). In order to confirm that the non-lytic phenotype of *L. lactis* Δ*ftsH* was not due to an hypothetical requirement of a functional FtsH for holin triggering, MitC-induced *L. lactis* TP712 lysogens were treated with the ionophores valinomycin, nigericin, and nisin, which dissipate the membrane potential (ΔΨ), the pH gradient (ΔpH) or both components of the proton motive force, respectively. In this way, ionophores will mimic the activity of the holin (i.e., loss of membrane integrity) and lysis will proceed normally even in the hypothetical case of a non-functional holin in *L. lactis ΔftsH*.

**FIGURE 2:**
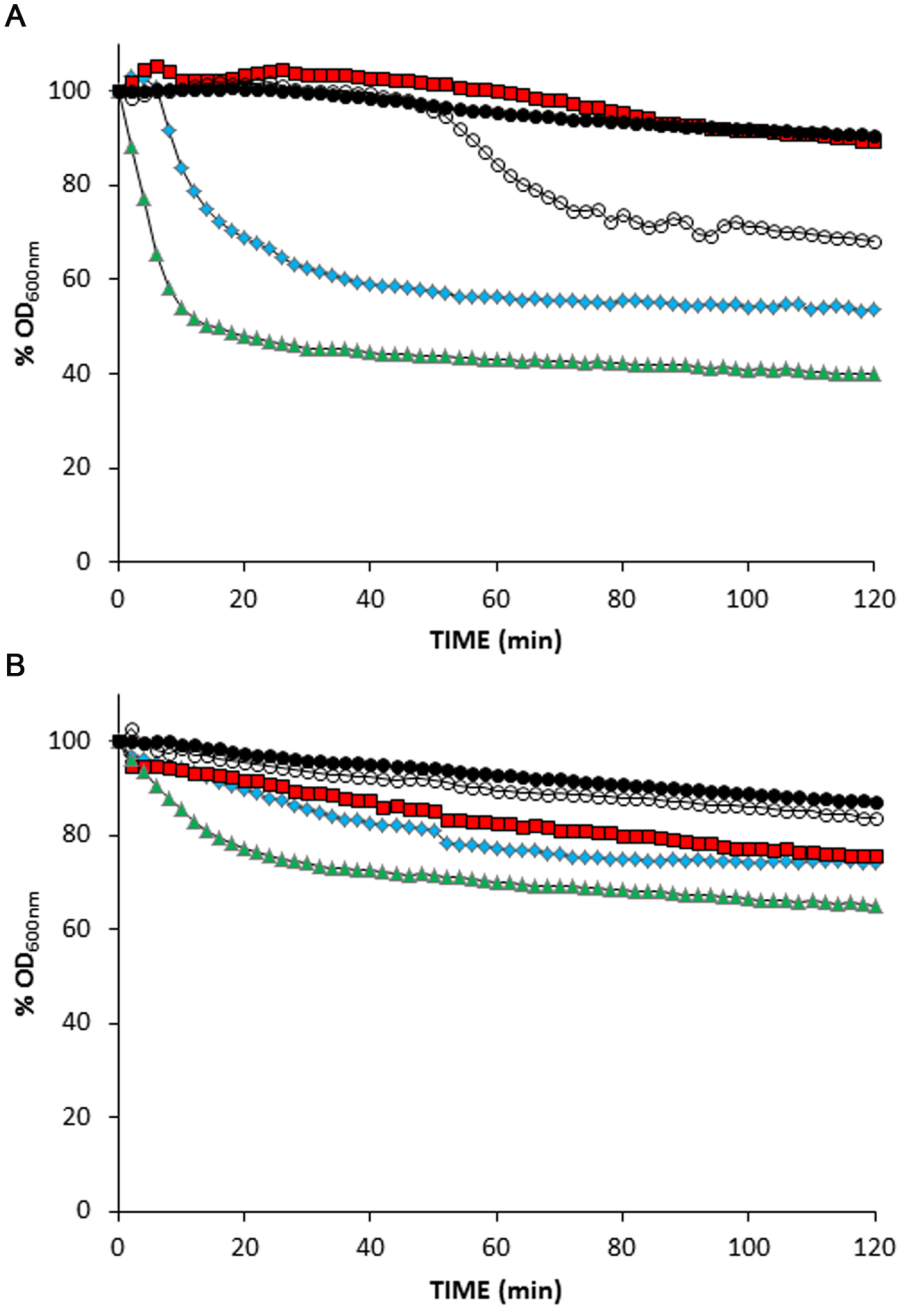
**Treatment of MitC-induced *L. lactis* lysogens with ionophores. (A)** *L. lactis* UKLc10 TP712 (wildtype). **(B)** *L. lactis* UKLc10 Δ*ftsH* TP712. After 60 min of induction with MitC (time 0), cell suspensions were treated with 1 μM of valinomycin (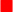), nigericin (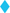), and nisin (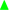) or non-treated (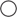). Cells from non-MitC induced cultures were also treated with ionophores but lysis did not occur and only data of nisin addition are shown for clarity (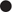). The OD_600nm_ of the cell suspensions just before adding the ionophores was considered 100%. The experiment was carried out in triplicate and results from a representative experiment are shown.

Without the addition of ionophores, suspensions of wildtype TP712 lysogenic cells, prepared after 60 min of MitC induction, started lysing roughly after 35 min of incubation (**Figure 2A**) that fits well with the 90 min onset of lysis previously observed in TP712 lysogens induced with MitC (Roces et al., 2013). On the contrary, lysis was immediately triggered by nisin and nigericin, while valinomycin appeared to inhibit it (**Figure 2A**). Control cells which were not induced with MitC did not lyse after the addition of the ionophores (**Figure 2** and data not shown), confirming that the observed lysis was not due to any secondary lytic effects of the ionophores themselves, for example due to activation of bacterial autolysins.

*Lactococcus lactis* Δ*ftsH* TP712 lysogens behaved differently and the treatment with ionophores did not prompt significant lysis (**Figure 2B**). No lysis was detected in cell suspensions of MitC-induced cells nor did the control cells lyse after the addition of the ionophores. Only nisin caused some lysis that accounted for 30%, half of that achieved with wildtype cells under the same experimental conditions. In summary, these results suggest that holin malfunctioning and lack of membrane depolarization is not behind the lack of lysis in *L. lactis* Δ*ftsH*.

### The Endolysin LysTP712 is Synthesized and Active in *L. lactis* Δ*ftsH*

The other player required to lyse host cells is the endolysin of TP712 responsible for cleavage of the peptidoglycan. l*ysTP712* (orf56) is translationally coupled with *hol*TP712 by overlapping start and stop codons, respectively. LysTP712 is 429 aa long and features the modular structure typical for endolysins from phages infecting Gram-positive bacteria. At its N-terminus it has a catalytic domain (CD; residues 33–216) that belongs to the glycosyl hydrolase family 25 (GH_25) (Pfam PF01183) giving it lysozyme activity (EC 3.2.1.17). The CBD resides in the C-terminus and is composed of two tandem LysM (Pfam PF01476) motifs (residues 333–374 and 387–427, respectively) (**Figure 1**). BlastP searches identified endolysins of the lactococcal phages phiAM2 (GenBank accession: AAG24366), ul36 (NP_663692), TP901-1 (NP_112716), phiLC3 (NP_996722), TPW22 (AAF12705), Tuc2009 (NP_108734), and P335 (ABI54253) as the closest homologs, all sharing the same architecture with amino acid identity level ranging from 83 to 96%. Similarity to endolysins of other phages infecting bacteria from the genera *Enterococcus, Oenococcus, Pediococcus*, and *Lactobacillus* were also found (identities ranging from 45 to 60%). These endolysins possess the same CD albeit linked to other CBDs. According to the recent analysis by Oliveira et al. (2013), a putative N-terminal signal peptide appears to be present in LysTP712, which shares features of a possible SAR domain.

**FIGURE 3:**
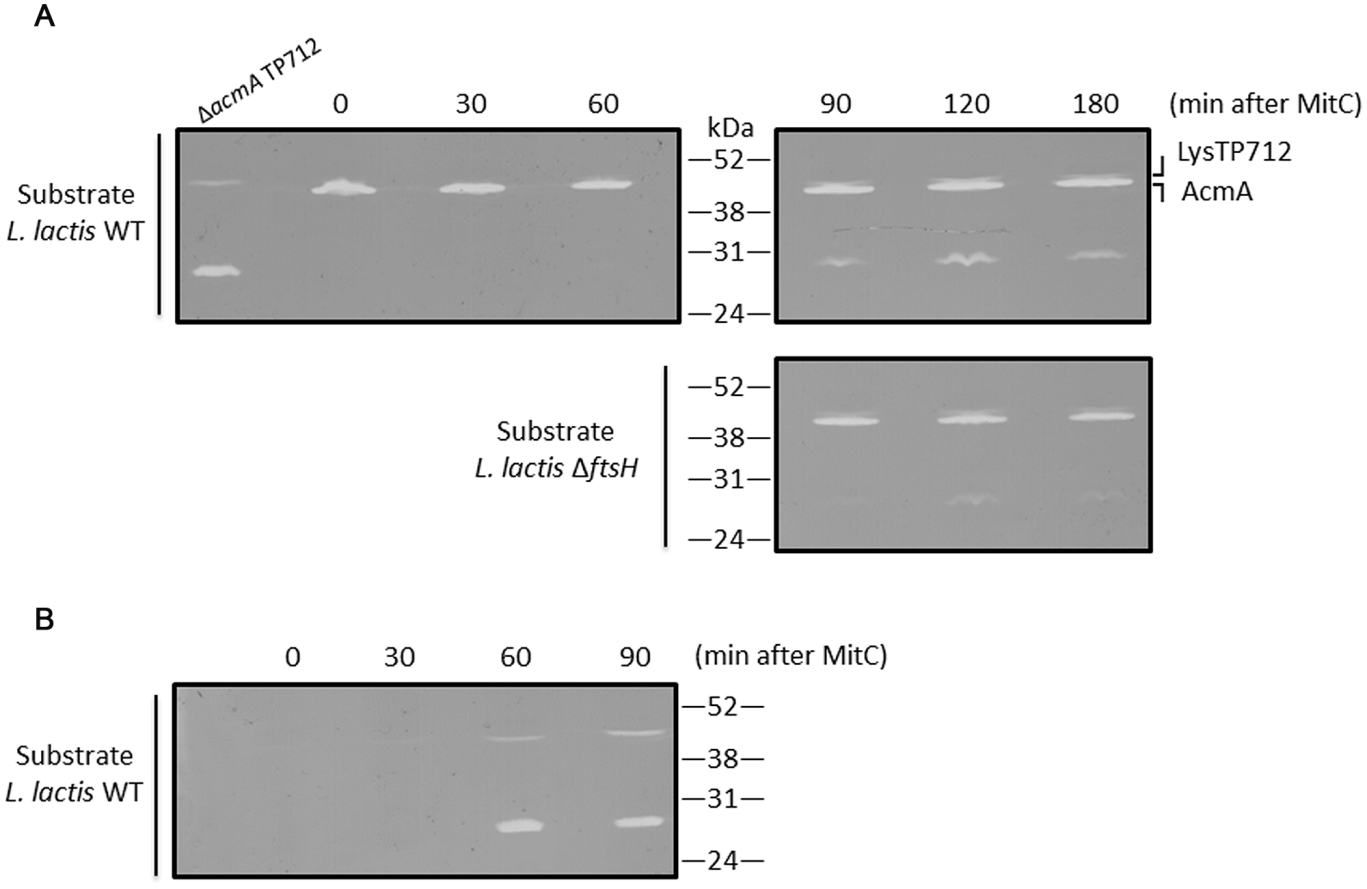
**Detection of LysTP712 in zymograms. (A)** *L. lactis* Δ*ftsH* TP712 was induced with MitC and samples were taken at the indicated times. Total protein extracts were run on 10% SDS-PAGE gels containing autoclaved *L. lactis* NZ9000 (wildtype, WT) or *L. lactis* Δ*ftsH* cells as substrate. **(B)** A *L. lactis* TP712 lysogen lacking the major autolysin AcmA (Δ*acmA* TP712) was also induced for 90 min and used to identify LysTP712 without the interference of AcmA.

The first question to answer was if LysTP712 could be synthesized in *L. lactis* Δ*ftsH*. To this end, total protein extracts from TP712 lysogens induced with MitC were analyzed in zymograms to reveal the peptidoglycan hydrolytic activity of the endolysin. As shown in **Figure 3A**, a lytic band with the expected size (46.5 kDa) was visible at 60 min after induction and become more noticeable over time. Due to the similar size of the endolysin to that of the major autolysin AcmA (46.6 kDa), *L. lactis* MG1363 Δ*acmA* was lysogenized with TP712 and protein extracts from MitC-induced cultures were run in zymograms to be able to unequivocally identify the lytic activity of LysTP712 over that of the autolysin. Activity of LysTP712 was already observed as a very faint band after 30 min of induction and become evident at 60 min and onward (**Figure 3B**). Contrary to the *L. lactis* Δ*ftsH* lysogen, samples at later time points could not be handled because lysis of the culture. LysTP712 was also detected in protein extracts from other wildtype TP712 lysogens (Supplementary Figure S2).

On the other hand, zymograms prepared with *L. lactis* Δ*ftsH* cells as substrate also yielded clearing zones demonstrating that LysTP712 was also active *in vitro* against *L. lactis* Δ*ftsH* (**Figure 3**).

Another lytic activity of about 27–29 kDa was also revealed in the zymograms at the time as LysTP712 detection (**Figure 3**). Attempts to identify this protein by eluting it from the gel followed by mass fingerprinting failed. Only host proteins and the phage anti-repressor were identified and the protein responsible of this lytic activity remains to be identified. Nevertheless, this band was present in both *L. lactis* Δ*ftsH* and wildtype TP712 lysogens and could not be responsible for the non-lytic phenotype of *L. lactis* Δ*ftsH*. Neither this hydrolytic activity nor LysTP712 were ever observed in protein extracts from non-lysogens treated with MitC (Supplementary Figure S2).

### Binding of the CBD of LysTP712 to *L. lactis* Δ*ftsH* is Reduced

The 2xLysM CBD of LysTP712 was fused to the C-terminus of the red fluorescent protein mCherry to monitor its binding to lactococcal cells. The reasoning behind this was that, even though LysTP712 was synthesized in – and enzymatically active against *L. lactis* mutant cells lacking FtsH, LysTP712 might be delocalized from its site of action or even unable to bind to its substrate.

**FIGURE 4:**
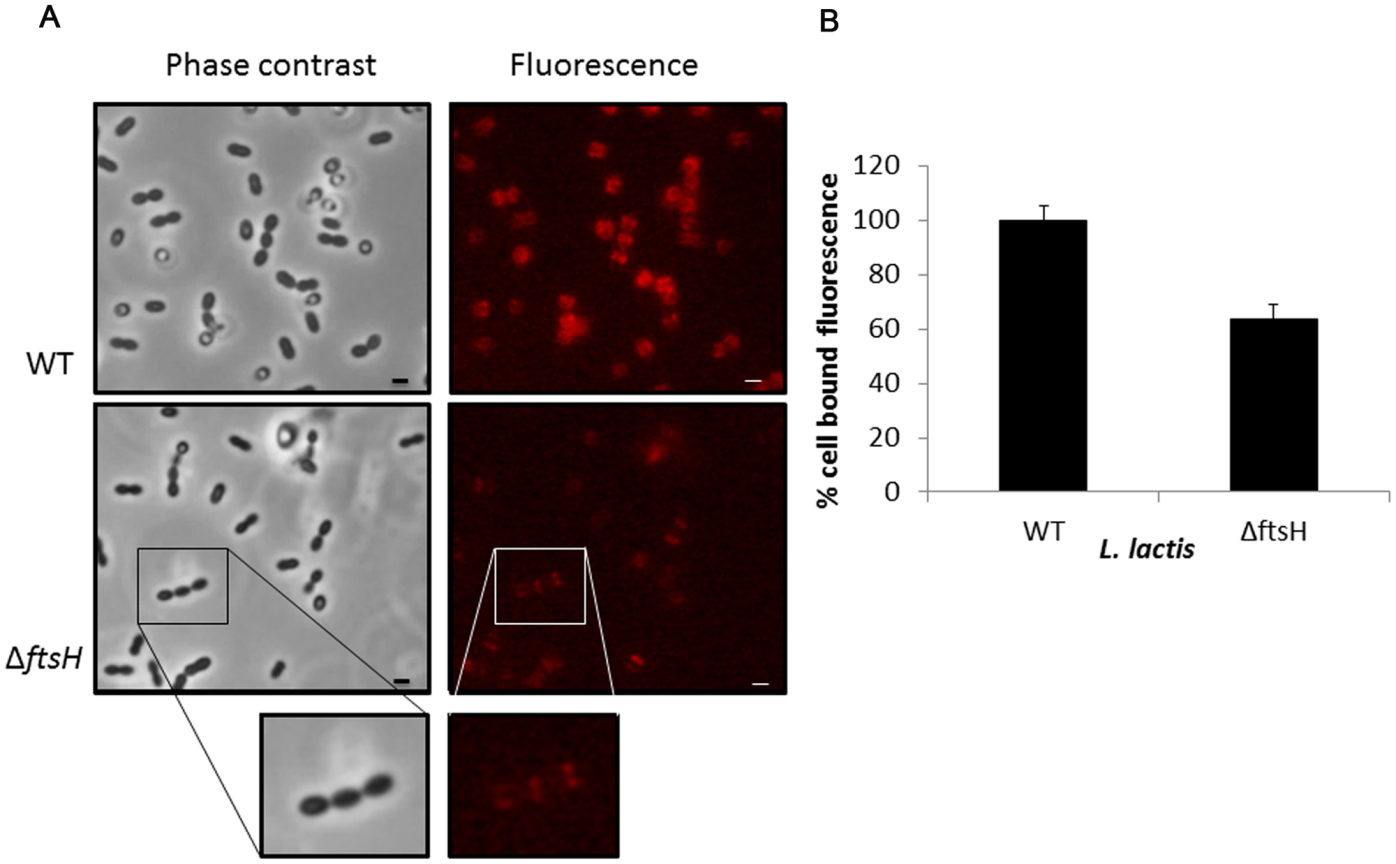
**Binding of the fluorescent labeled 2xLysM cell binding domain of LysTP712 to *L. lactis*. (A)** *L. lactis* NZ9000 (wildtype, WT) and *L. lactis* Δ*ftsH* cells under phase contrast (left panels) and epifluorescence microscopy (right panels) after incubation with mCherry-2xLysM_TP712_. Scale bar: 1 μm. **(B)** Fluorometer measurements. Cell-bound fluorescence of *L. lactis* NZ9000 (wildtype, WT) was taken as 100%.

Exponentially growing cells of *L. lactis* NZ9000 and *L. lactis* Δ*ftsH* were collected and mixed with the recombinant mCherry-2xLysM_TP712_ fluorescent protein and subsequently inspected under the fluorescent microscope. The reporter protein localized at the septa of wildtype cells where peptidoglycan is being actively synthesized (**Figure 4**). A similar pattern was seen on *L. lactis* Δ*ftsH* cells but fluorescence was qualitatively less intense and hardly detectable on some cells (**Figure 4**). No signal at all was detected when using cells from the unrelated species S*taphylococcus xylosus* CTC1642 (data not shown), in line with the species-specific interaction of endolysins with their own substrates.

Quantification of the fluorescent signal bound to *L. lactis* cells using a fluorometer demonstrated that binding of the reporter protein was compromised in *L. lactis* Δ*ftsH* (**Figure 4**). Compared to the wildtype, *L. lactis* Δ*ftsH* cells bound 40% less protein (*p* < 0.05), in agreement with the lower intensity observed under the microscope, providing strong evidence that the lack of lysis in *L. lactis* Δ*ftsH* could be due to a reduced ability of the phage endolysin to bind to its cell wall target.

## Discussion

In this work we endeavored to explain the phage resistant phenotype of *L. lactis* devoid of the membrane protease FtsH. As described previously, phage-infected *L. lactis* Δ*ftsH* cells did not lyse despite the accumulation of infective phages in the cytoplasm (Roces et al., 2013). Information about the functions of FtsH in lactic acid bacteria and, particularly, in *Lactococcus* is scarce. Contrary to *E. coli*, *ftsH* is dispensable in *L. lactis*, like in several other Gram-positives, and null mutants are viable (Nilsson et al., 1994; Pinto et al., 2011; Roces et al., 2013). However, these mutants may display pleiotropic effects, particularly under stress conditions considering that FtsH belongs to the cell envelope stress response of *L. lactis* (Martínez et al., 2007). So far, FtsH has not ever been described as being involved in phage propagation, besides its role in regulating the lytic/lysogeny switch in phage dddd(Langklotz et al., 2012).

The closer inspection of the TP712 lytic cassette revealed similarities to the antiholin and holin proteins of phage 21 in terms of structure, i.e., two TMDs, presence of extra positive charges at the antiholin N-terminus and a dual start motif within the holin gene. The delay in translocating TMD1, which is required to trigger holin, correlates with the number of positive charges. Indeed, the addition of just two more positive residues to S^21^68 already blocks translocation (Park et al., 2006). Thus, the presence of seven positive charges at the N-terminus of Hol88TP712 led us to consider that proteolytic processing by FtsH might be needed to tune the timing of lysis in TP712. Two lines of evidence demonstrated that this was not the case. Firstly, proteolysis of the full-length Hol88TP712 in wildtype cells did not occur when the holin gene was expressed from a plasmid. Secondly, and more revealing, *L. lactis* Δ*ftsH* did not lyse even after mimicking holin activity using ionophores (see **Figure 2**). This was in contrast to wildtype lysogens that immediately lysed after the treatment demonstrating that, at least after 60 min of prophage induction, the phage endolysin was ready to be activated and ionophores were indeed able to mimic the holin lesions under the experimental conditions, overall suggesting that lack of lysis of *L. lactis* Δ*ftsH* was not due to holin malfunctioning. The fact that not all the ionophores had the same lytic outcome might be related to their different ion selectivity. Lysis was preferentially induced by disruption of the ΔpH gradient (i.e., nigericin) rather than the membrane potential as shown by the absence of lysis after addition of valinomycin, highly selective for potassium ions. Nisin, able to disrupt both components of the proton motive force, was the most effective in inducing lysis. Nisin has already been reported as the best membrane depolarizer to induce lysis mediated by Lys44, the endolysin of the *O. oeni* phage fOg44 (Nascimento et al., 2008) and also acts synergistically with the staphylococcal endolysin LysH5 (García et al., 2010). The biochemistry behind these observations remains to be elucidated but, in any case, *L. lactis* Δ*ftsH* cells did not lyse to the same extent as the wildtype cells did, suggesting a defective endolysin activity.

Lack of lysis of *L. lactis* Δ*ftsH* lysogens might be due to a putative requirement of FtsH to activate LysTP712, for example, by processing its N-terminus. However, zymograms revealed that synthesis of the endolysin was not impaired in *L. lactis* Δ*ftsH* lysogens and that the protein was active against *L. lactis* Δ*ftsH* cells. Unfortunately, we were unable to quantify LysTP712 enzymatic activity, because we did not find the right experimental conditions to measure lysis in cell suspensions (turbidity assays) with recombinant LysTP712 produced in *E. coli.* Lysis-from-without was never achieved and wildtype cells did not lyse even in the presence of nisin and nigericin (unpublished data). This is not surprising because some endolysins may require specific ions, ionic strength, etc. to achieve lysis-from-without *in vitro* and turbidity assays might not be sensitive enough (Nelson et al., 2012). Nevertheless, the zymograms run with cell extracts from *L. lactis* Δ*ftsH* lysogens matched those from wildtype cells, suggesting that at least the endolysin was properly synthesized and active *in vitro* against *L. lactis*, regardless of the genetic background of the cells used as substrate. It should be noticed in any case that localization of LysTP712 within the cell could not be properly ascertained as the protein was only detected in the insoluble fraction after breaking the cells. Therefore, a putative role of FtsH in placing LysTP712 in the cell wall compartment for proper activity cannot be ruled out.

Zymograms also revealed the presence of an additional lytic activity in wildtype and Δ*ftsH* TP712 lysogens. This activity must be encoded by the phage itself or is induced during the propagation of TP712 as it is only present in TP712 lysogens and follows the same synthesis pattern as LysTP712. The recent description of multimeric endolysins (Nelson et al., 2006; Dunne et al., 2014; Proenca et al., 2015) opens the question if that is the case of LysTP712 and remains to be studied. Yet, as judged by the zymograms from MitC-induced cultures, the presence/absence of FtsH does not alter the pattern of the lytic bands.

Fluorescence-based assays demonstrated that the LysTP712 CBD bound less efficiently to *L. lactis* Δ*ftsH* cells. The binding pattern of 2xLysM_TP712_ at the septum is in agreement with the previously described localization of AcmA, a LysM-containing *L. lactis* autolysin (Steen et al., 2003). The bacterial LysM domain has been shown to recognize *N*-acetyl-glucosamine-*X-N*-acetyl-glucosamine polymers, including bacterial peptidoglycan (Mesnage et al., 2014). Its affinity for the substrate is modulated by several factors including the nature of the stem peptide and the composition of the lipoteichoic acids present in the cell wall (Steen et al., 2008; Mesnage et al., 2014) while modifications such as *O*-acetylation and *N*-deacetylation of the peptidoglycan do not affect LysM-binding to *L. lactis* cells (Meyrand et al., 2007; Veiga et al., 2007). Reduced binding of the endolysin to its substrate implies a less efficient lysis of the host and may consequently explain, at least in part, the non-lytic phenotype of *L. lactis* Δ*ftsH* TP712 lysogens. Similarly, a decrease in autolysis has been correlated to a reduce binding of the autolysin AcmA (Steen et al., 2008).

In summary, we have shown that FtsH is not directly involved in regulating or triggering the lytic step of TP712. Our results strongly suggest that the non-lytic phenotype of *L. lactis* Δ*ftsH* is determined, at least in part, by the reduced binding of the phage endolysin to its substrate. These results support a putative role of FtsH in modulating the architecture of the cell envelope and it is fair to consider that changes at the surface may also have an impact on the catalytic activity of the phage endolysin. This observation is further strengthened by recent reports showing that *Lactobacillus plantarum* mutants lacking FtsH display altered cell surface properties (Bove et al., 2012). On these premises, further characterization of the composition of the cell envelope of *L. lactis* Δ*ftsH* and putative FtsH targets is currently in progress.

## Author Contributions

CR, AC, and SE performed the experiments and contributed to the analysis of data. UW, PG, AR, and BM conceived the work and contributed to design of the work, analysis, and interpretation of the data. CR, UW, BM drafted the manuscript. All authors approved the final version of the submitted manuscript.

## Conflict of Interest Statement

The authors declare that the research was conducted in the absence of any commercial or financial relationships that could be construed as a potential conflict of interest.
