# Supplementary figures and images for: Reduced Binding of the Endolysin LysTP712 to Lactococcus lactis ΔftsH Contributes to Phage Resistance
Source: Front Microbiol. 2016 Feb 11;7:138. doi: 10.3389/fmicb.2016.00138 (PMC4749879; doi:10.3389/fmicb.2016.00138)

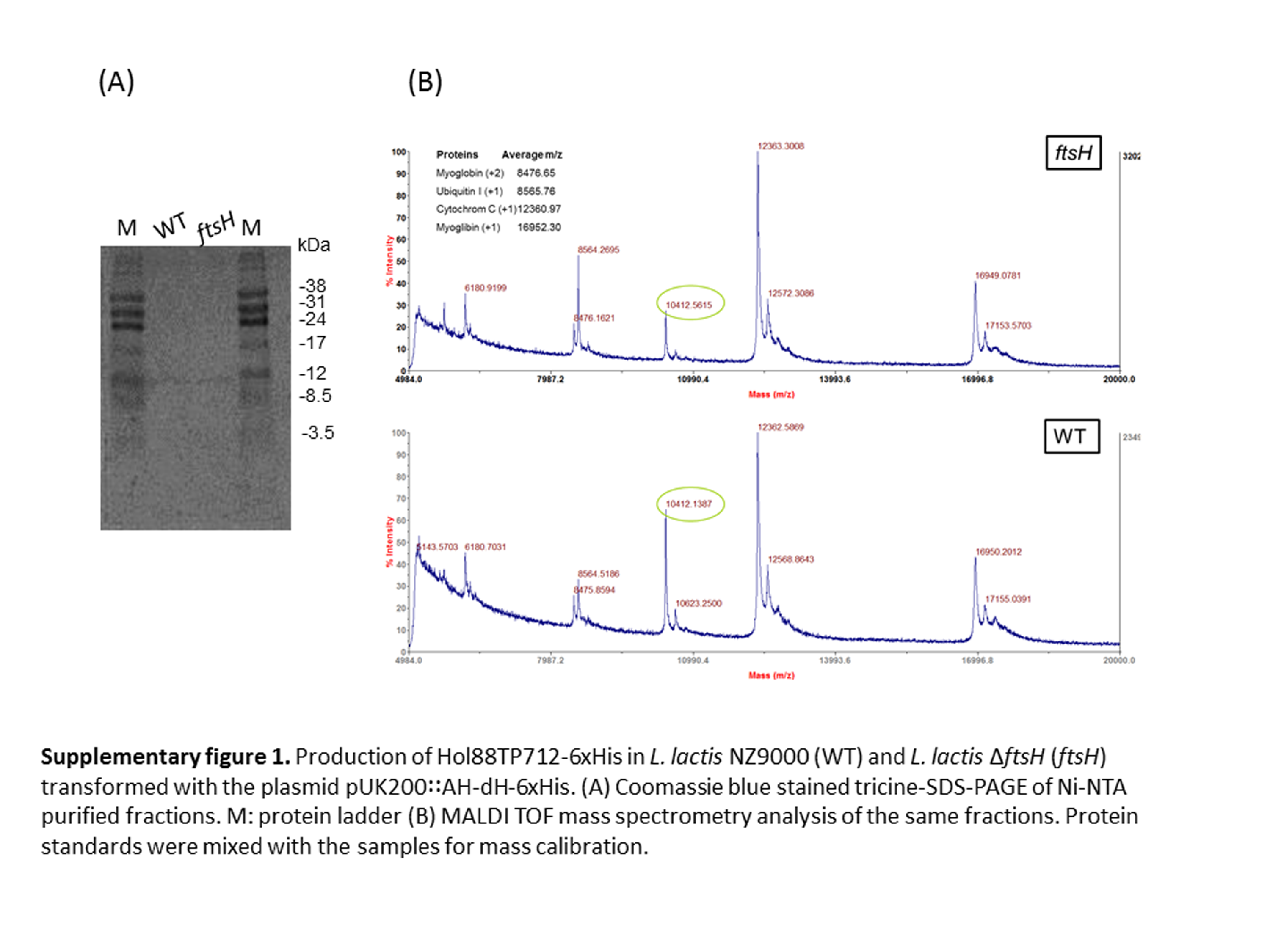

Supplement: Supplementary file 1 [file Image_1.TIF]
