# Supplementary material for: Reduced Binding of the Endolysin LysTP712 to Lactococcus lactis ΔftsH Contributes to Phage Resistance
Source: Front Microbiol. 2016 Feb 11;7:138. doi: 10.3389/fmicb.2016.00138 (PMC4749879; doi:10.3389/fmicb.2016.00138)
Supplement: Supplementary file 2 [file Image_2.PDF]

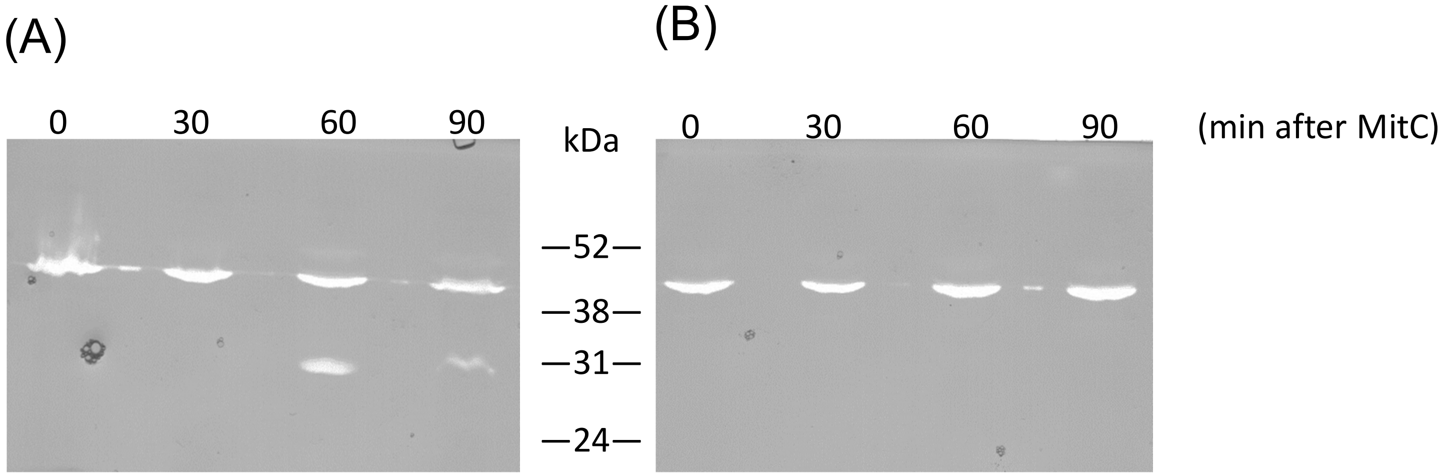

Supplementary Figure 2. Wildtype *L. lactis* MG1363 TP712 lysogen (A) and *L. lactis* MG1363 (B) were induced with MitC and samples were taken at the indicated times. Total protein extracts were run on 10 % SDS-PAGE gels containing autoclaved *L. lactis* NZ9000 cells.
